# Bis[μ-pentane-2,4-dionato(1−)]bis­{aqua­[1,1,1,5,5,5-hexa­fluoro­pentane-2,4-dionato(1−)]cobalt(II)}

**DOI:** 10.1107/S1600536809044389

**Published:** 2009-10-31

**Authors:** Gerald O. Hunter, Matthias Zeller, Brian D. Leskiw

**Affiliations:** a Youngstown State University, Department of Chemistry, 1 University Plaza, Youngstown, OH 44555, USA

## Abstract

The title complex, [Co_2_(C_5_HF_6_O_2_)_2_(C_5_H_7_O_2_)_2_(H_2_O)_2_], is centrosymmetric with a crystallographic inversion center in the middle of the mol­ecule. The octa­hedrally coordinated Co^II^ atoms are bridged by two chelating acetyl­acetonate (acac) ligands and two more electron-poor 1,1,1,5,5,5-hexa­fluoro­pentane-2,4-dionato (hfac) ligands are bonded terminally in a solely chelating manner. The coordinated water mol­ecules form inter­molecular O—H⋯O hydrogen bonds with electron-rich acac O atoms of neighboring mol­ecules, leading to strings of mol­ecules along the *a* axis.

## Related literature

For mass spectrometry of β-diketonates, see: Reichert & Westmore (1969 ▶); Westmore (1976 ▶); Lerach & Leskiw (2008 ▶). For applications of β-diketonate complexes, see: Condorelli *et al.* (2007 ▶); Silvennoinen *et al.* (2007 ▶); Fahlmen (2006 ▶). For related structures, see: Hunter *et al.* (2009*a* ▶,*b* ▶); Lerach *et al.* (2007 ▶); Cotton & Elder (1966 ▶); McCann *et al.* (2001 ▶).
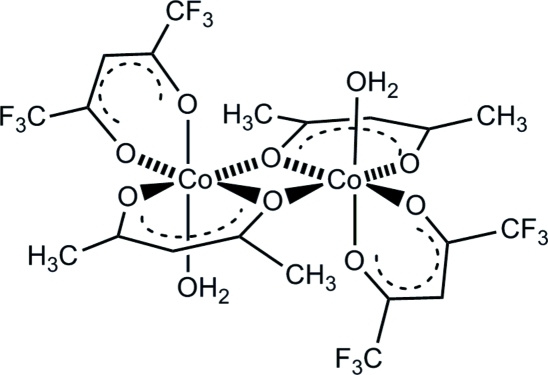


## Experimental

### 

#### Crystal data

[Co_2_(C_5_HF_6_O_2_)_2_(C_5_H_7_O_2_)_2_(H_2_O)_2_]
*M* *_r_* = 766.22
Triclinic, 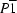

*a* = 7.563 (3) Å
*b* = 9.541 (4) Å
*c* = 9.716 (4) Å
α = 94.865 (6)°
β = 92.792 (6)°
γ = 93.622 (6)°
*V* = 696.2 (5) Å^3^
*Z* = 1
Mo *K*α radiation
μ = 1.32 mm^−1^
*T* = 100 K
0.20 × 0.16 × 0.08 mm

#### Data collection

Bruker APEXII CCD diffractometer
Absorption correction: multi-scan (*SADABS*; Bruker, 2008 ▶) *T*_min_ = 0.717, *T*_max_ = 0.900
6687 measured reflections
3378 independent reflections
1937 reflections with *I* > 2σ(*I*)
*R*_int_ = 0.073

#### Refinement

*R*[*F* ^2^ > 2σ(*F* ^2^)] = 0.058
*wR*(*F* ^2^) = 0.119
*S* = 0.97
3378 reflections
207 parameters
2 restraints
H atoms treated by a mixture of independent and constrained refinement
Δρ_max_ = 0.63 e Å^−3^
Δρ_min_ = −0.73 e Å^−3^

### 

Data collection: *APEX2* (Bruker, 2008 ▶); cell refinement: *SAINT* (Bruker, 2008 ▶); data reduction: *SAINT*; program(s) used to solve structure: *SHELXTL* (Sheldrick, 2008 ▶); program(s) used to refine structure: *SHELXTL*; molecular graphics: *SHELXTL*; software used to prepare material for publication: *SHELXTL*.

## Supplementary Material

Crystal structure: contains datablocks I, global. DOI: 10.1107/S1600536809044389/lx2118sup1.cif

Structure factors: contains datablocks I. DOI: 10.1107/S1600536809044389/lx2118Isup2.hkl

Additional supplementary materials:  crystallographic information; 3D view; checkCIF report

## Figures and Tables

**Table 1:** Hydrogen-bond geometry (Å, °)

*D*—H⋯*A*	*D*—H	H⋯*A*	*D*⋯*A*	*D*—H⋯*A*
O5—H5*A*⋯O2^i^	0.83 (4)	2.25 (3)	2.973 (4)	147 (5)
O5—H5*B*⋯O3^ii^	0.84 (2)	1.87 (2)	2.703 (4)	169 (5)

Symmetry codes: (i) 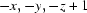; (ii) 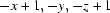.

**Fig. 1.**
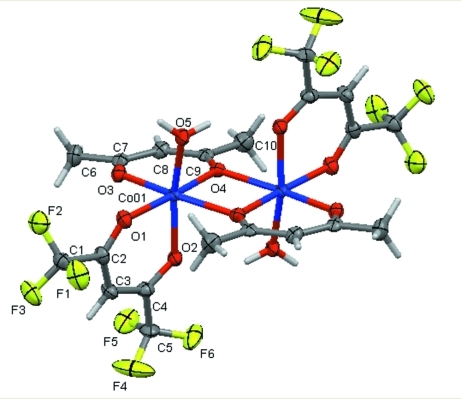
ORTEP representation of the title compound (50% probability displacement ellipsoids)

**Fig. 2.**
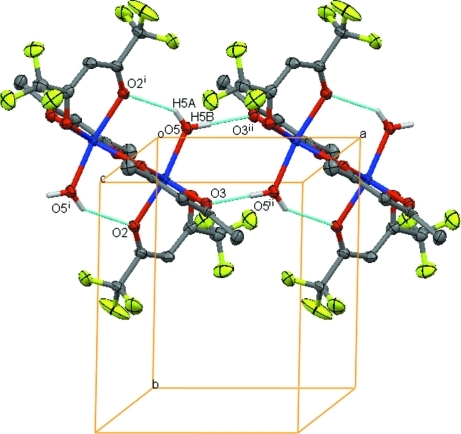
View of a section of one of the hydrogen bonded chains along the a axis. Hydrogen bonds are symbolized by blue dashed lines. Symmetry codes: (i) -x, -y, -z + 1; (ii) -x + 1, -y, -z + 1.

**Fig. 3.**
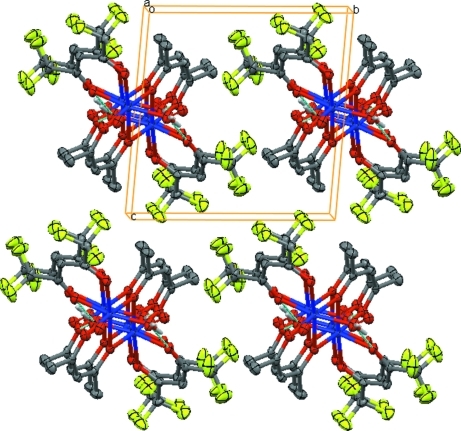
Packing view of the title structure, view down the a axis. Hydrogen bonds are symbolized by blue dashed lines.

## supplementary crystallographic information

### Comment

Our interest in β-diketonate complexes, specifically fluorinated acetylacetonate (acac) derivatives, stems from their volatility and most notably their ability to undergo both partial and complete ligand exchange reactions. Recent applications of β-diketonate complexes can be seen in the areas of catalysis and microelectronics (Silvennoinen *et al.* 2007), and the deposition of metallic or ceramic thin films (Condorelli *et al.* 2007). Furthermore, β-diketonates are ideally suited as precursors for vapor deposition processes (Fahlmen 2006). Research in the area of ligand exchange reactions is being conducted with the goal of observing gas-phase reactions and ligand exchange *via* mass spectrometry (Lerach & Leskiw 2008).

In previous structure reports on complexes with the 1,1,1-trifluoro-5,5-dimethyl-2,4-hexanedione (tftm) ligand, we reported three monometallic complexes with zinc, nickel and cobalt as the metal (Hunter *et al.* 2009*a* and 2009*b*, Lerach *et al.* 2007). Using a mixture of two acetylacetonate ligands, parent pentane-2,4-dionate (acac) and the hexafluoro derivative 1,1,1,5,5,5-hexafluoropentane-2,4-dionate (hfac), the title compound was obtained as a dimeric biscobalt complex after purification by sublimation and recrystallization (Figure 1). The complex is centrosymmetric with the center of the complex being located on a crystallographic inversion center, and each of the metal centers exhibits an only slightly distorted octahedral coordination environment of six oxygen atoms as expected for Co(II) complexes. The ligand environment of each metal center is composed of a chelating hfac ligand, one coordinated water molecule and two chelating bridging acac ligands. The connection between the two metal ions is facilitated by the two µ-oxygen atoms from these two acac ligands. The coordination modes of the two types of β-diketonate ligands are thus quite distinct based on the electron densities available at the oxygen atoms of the ligands. The less electron rich hfac ligand is a chelating terminal ligand, the more electron rich acac ligands are chelating and bridging between the metal centers. One of the oxygen atoms of the hfac ligand is *trans* to the coordinated water molecule, the other *trans* to the bridging acac µ-O4 atom.

The general motif for this dimeric structure is not unknown. For cobalt, dimeric complexes similar to the title compound were for example reported with only acetyl acetonate as the ligand rather than two different acac derivatives. Structures are known for bis(aqua-(µ^2^-pentane-2,4-dionato-*O*,*O*,*O*')-(pentane-2,4-dionato)-cobalt(II)) itself (Cotton & Elder, 1966) (but the quality of the structure is very low) and as a co-crystal with tetra-aqua-(acetylacetonato)-cobalt(II) perchlorate (McCann *et al.*, 2001). In both structures the dimeric complexes exhibit the same centrosymmetric structural motif with the same coordination arrangement of acac and water ligands as in the title compound. The metal-oxygen bonding distances in the title compound and the well resolved structure are the same within 0.04 Å.

The coordinated water molecules are involved in hydrogen bonding interactions (Table 1). An intramolecular hydrogen bond stabilizes the dimeric structure in both the title structure and the acac parent complex. In the title compound these hydrogen bonds are oriented towards the neighboring oxygen atom O2^i^ of the hfac ligand (symmetry operator (i): -*x*, -*y*, -*z* + 1). The other H atom of the water molecule makes a strong intermolecular H bond to O3^ii^ in a neighboring molecule (symmetry operator (ii): -*x* + 1, -*y*, -*z* + 1). The intermolecular hydrogen bonds are arranged in inversion symmetric pairs that connect molecules along the *a*-axis leading to strongly hydrogen bonded strings of molecules along that axis (Figure 2). Individual interactions between these strings of molecules, on the other hand, are weak and are mostly based on shape recognition of the acac and hfac ligands (Figure 3).

It should be stated that this oxygen atom O3 is probably the most electron rich in the dimer (being an acac O atom and not bridging) and the O—H···O hydrogen bond formed is thus the strongest one possible in this system. It could therefore be assumed that the packing of the molecules is at least partially based on the ability to form this strong hydrogen bond (rather than a weaker one towards one of the less electron rich O atoms). This is however at least partially ruled out by the fact that the acac-only complex (Cotton & Elder, 1966) adopts the same hydrogen bonding motif with infinite hydrogen bonded chains where the hydrogen bonding acceptor is the monodentate oxygen atom of the bridging acac ligand. Other influences than only the electron donor ability of hydrogen atom acceptor thus must play an important role as well, which might be found among the ability to form pairwise hydrogen bonds, shape recognition between the molecules, or preassembly of hydrogen bonded chains in solution before crystallization.

### Experimental

The synthesis of the title compound involved equal molar concentrations (1.0 mmol) of both cobalt acetylacetonate and hexafluoroacetylacetonate, dissolved in concentrated methanol, and being reacted under a steady reflux for forty-eight hours. The solvent was subsequently removed *in vacuo*, and the desired product was purified *via* sublimation under vacuum. The desired product was re-crystallized overnight by vapor diffusion of hexanes into a solution of diethyl ether.

### Refinement

The water H atoms were located in a difference density Fourier map. The O—H distances were restrained to 0.84 (2) Å. All other H atoms were placed in calculated positions with C—H distances of 0.98 (methyl) and 0.95 Å (CH). The methyl and hydroxyl H's were refined with an isotropic displacement parameter *U*_iso_ of 1.5 times *U*_eq_ of the adjacent carbon or oxygen atom, and the C—H hydrogen atom with *U*_iso_ = 1.2 *U*_eq_(C). Methyl hydrogen atoms were allowed to rotate to best fit the experimental electron density.

### Crystal data

**Table:**

[Co_2_(C_5_HF_6_O_2_)_2_(C_5_H_7_O_2_)_2_(H_2_O)_2_]	*Z* = 1
*M**_r_* = 766.22	*F*(000) = 382
Triclinic, *P*1	*D*_x_ = 1.828 Mg m^−^^3^
Hall symbol: -P 1	Mo *K*α radiation, λ = 0.71073 Å
*a* = 7.563 (3) Å	Cell parameters from 1148 reflections
*b* = 9.541 (4) Å	θ = 2.7–30.6°
*c* = 9.716 (4) Å	µ = 1.32 mm^−^^1^
α = 94.865 (6)°	*T* = 100 K
β = 92.792 (6)°	Block, red
γ = 93.622 (6)°	0.20 × 0.16 × 0.08 mm
*V* = 696.2 (5) Å^3^	

### Data collection

**Table:**

Bruker APEXII CCD diffractometer	3378 independent reflections
Radiation source: fine-focus sealed tube	1937 reflections with *I* > 2σ(*I*)
graphite	*R*_int_ = 0.073
ω scans	θ_max_ = 28.3°, θ_min_ = 2.1°
Absorption correction: multi-scan (*SADABS*; Bruker, 2008)	*h* = −9→10
*T*_min_ = 0.717, *T*_max_ = 0.900	*k* = −12→12
6687 measured reflections	*l* = −12→12

### Refinement

**Table:**

Refinement on *F*^2^	Primary atom site location: structure-invariant direct methods
Least-squares matrix: full	Secondary atom site location: difference Fourier map
*R*[*F*^2^ > 2σ(*F*^2^)] = 0.058	Hydrogen site location: difference Fourier map
*wR*(*F*^2^) = 0.119	H atoms treated by a mixture of independent and constrained refinement
*S* = 0.97	*w* = 1/[σ^2^(*F*_o_^2^) + (0.0425*P*)^2^] where *P* = (*F*_o_^2^ + 2*F*_c_^2^)/3
3378 reflections	(Δ/σ)_max_ < 0.001
207 parameters	Δρ_max_ = 0.63 e Å^−^^3^
2 restraints	Δρ_min_ = −0.72 e Å^−^^3^

### Special details

**Table:**

Geometry. All e.s.d.'s (except the e.s.d. in the dihedral angle between two l.s. planes) are estimated using the full covariance matrix. The cell e.s.d.'s are taken into account individually in the estimation of e.s.d.'s in distances, angles and torsion angles; correlations between e.s.d.'s in cell parameters are only used when they are defined by crystal symmetry. An approximate (isotropic) treatment of cell e.s.d.'s is used for estimating e.s.d.'s involving l.s. planes.
Refinement. Refinement of *F*^2^ against ALL reflections. The weighted *R*-factor *wR* and goodness of fit *S* are based on *F*^2^, conventional *R*-factors *R* are based on *F*, with *F* set to zero for negative *F*^2^. The threshold expression of *F*^2^ > σ(*F*^2^) is used only for calculating *R*-factors(gt) *etc*. and is not relevant to the choice of reflections for refinement. *R*-factors based on *F*^2^ are statistically about twice as large as those based on *F*, and *R*- factors based on ALL data will be even larger.

### Fractional atomic coordinates and isotropic or equivalent isotropic displacement parameters (Å^2^)

**Table:**

	*x*	*y*	*z*	*U*_iso_*/*U*_eq_	
C1	0.5543 (6)	0.2269 (5)	0.8934 (5)	0.0302 (11)	
C2	0.3920 (6)	0.2188 (5)	0.7936 (5)	0.0242 (10)	
C3	0.2836 (6)	0.3329 (5)	0.7993 (5)	0.0259 (11)	
H3	0.3165	0.4122	0.8634	0.031*	
C4	0.1302 (6)	0.3346 (5)	0.7153 (5)	0.0247 (10)	
C5	0.0245 (6)	0.4664 (5)	0.7323 (5)	0.0315 (12)	
C6	0.4853 (6)	0.3448 (5)	0.2998 (5)	0.0323 (12)	
H6A	0.5072	0.4195	0.3754	0.048*	
H6B	0.4443	0.3858	0.2157	0.048*	
H6C	0.5953	0.2986	0.2830	0.048*	
C7	0.3458 (5)	0.2382 (4)	0.3383 (5)	0.0228 (10)	
C8	0.1962 (5)	0.2021 (5)	0.2477 (5)	0.0236 (10)	
H8	0.1960	0.2434	0.1621	0.028*	
C9	0.0496 (5)	0.1142 (5)	0.2678 (5)	0.0212 (10)	
C10	−0.0963 (6)	0.0916 (5)	0.1565 (5)	0.0325 (12)	
H10A	−0.1091	−0.0083	0.1220	0.049*	
H10B	−0.0674	0.1487	0.0804	0.049*	
H10C	−0.2079	0.1196	0.1943	0.049*	
Co01	0.19236 (7)	0.06799 (6)	0.54981 (6)	0.01780 (18)	
F1	0.5216 (4)	0.1512 (3)	0.9985 (3)	0.0505 (9)	
F2	0.6953 (4)	0.1786 (3)	0.8342 (3)	0.0498 (9)	
F3	0.6031 (4)	0.3593 (3)	0.9485 (3)	0.0465 (8)	
F4	0.0423 (5)	0.5320 (4)	0.8555 (3)	0.0806 (13)	
F5	0.0781 (4)	0.5585 (3)	0.6446 (3)	0.0502 (9)	
F6	−0.1465 (4)	0.4379 (3)	0.7000 (4)	0.0579 (10)	
O1	0.3727 (4)	0.1059 (3)	0.7171 (3)	0.0241 (7)	
O2	0.0665 (4)	0.2433 (3)	0.6216 (3)	0.0229 (7)	
O3	0.3733 (4)	0.1857 (3)	0.4531 (3)	0.0243 (7)	
O4	0.0264 (3)	0.0478 (3)	0.3757 (3)	0.0198 (7)	
O5	0.2844 (4)	−0.1274 (3)	0.5039 (4)	0.0271 (8)	
H5A	0.214 (5)	−0.188 (4)	0.462 (5)	0.041*	
H5B	0.393 (3)	−0.143 (5)	0.506 (5)	0.041*	

### Atomic displacement parameters (Å^2^)

**Table:**

	*U*^11^	*U*^22^	*U*^33^	*U*^12^	*U*^13^	*U*^23^
C1	0.033 (3)	0.029 (3)	0.027 (3)	0.003 (2)	−0.006 (2)	−0.001 (2)
C2	0.025 (2)	0.026 (3)	0.021 (3)	−0.001 (2)	−0.004 (2)	0.004 (2)
C3	0.030 (2)	0.019 (3)	0.027 (3)	0.002 (2)	−0.004 (2)	−0.003 (2)
C4	0.028 (2)	0.018 (2)	0.028 (3)	0.005 (2)	0.005 (2)	0.002 (2)
C5	0.033 (3)	0.022 (3)	0.040 (3)	0.010 (2)	0.005 (2)	−0.006 (2)
C6	0.025 (2)	0.033 (3)	0.039 (3)	−0.003 (2)	0.001 (2)	0.009 (2)
C7	0.021 (2)	0.014 (2)	0.034 (3)	0.0067 (18)	0.005 (2)	0.002 (2)
C8	0.023 (2)	0.024 (3)	0.026 (3)	0.0076 (19)	0.001 (2)	0.008 (2)
C9	0.021 (2)	0.017 (2)	0.026 (3)	0.0105 (18)	−0.0004 (19)	−0.001 (2)
C10	0.033 (3)	0.039 (3)	0.027 (3)	0.005 (2)	−0.002 (2)	0.007 (2)
Co01	0.0150 (3)	0.0169 (3)	0.0214 (3)	0.0046 (2)	−0.0020 (2)	0.0003 (2)
F1	0.056 (2)	0.054 (2)	0.0414 (19)	−0.0057 (16)	−0.0206 (15)	0.0201 (17)
F2	0.0334 (16)	0.063 (2)	0.050 (2)	0.0093 (15)	−0.0129 (15)	−0.0097 (17)
F3	0.0498 (18)	0.0393 (19)	0.0461 (19)	−0.0049 (14)	−0.0192 (15)	−0.0035 (15)
F4	0.133 (3)	0.071 (3)	0.040 (2)	0.070 (2)	−0.013 (2)	−0.017 (2)
F5	0.0483 (19)	0.0323 (18)	0.075 (2)	0.0155 (14)	0.0118 (17)	0.0211 (17)
F6	0.0275 (16)	0.0332 (19)	0.115 (3)	0.0105 (13)	0.0148 (17)	0.0020 (19)
O1	0.0226 (16)	0.0200 (18)	0.0289 (18)	0.0034 (13)	−0.0072 (14)	0.0019 (15)
O2	0.0187 (15)	0.0201 (17)	0.0294 (18)	0.0041 (13)	−0.0019 (13)	−0.0006 (15)
O3	0.0184 (16)	0.0259 (18)	0.0290 (19)	0.0031 (13)	−0.0012 (14)	0.0040 (15)
O4	0.0199 (15)	0.0203 (17)	0.0194 (17)	0.0046 (13)	−0.0004 (13)	0.0016 (14)
O5	0.0153 (16)	0.0252 (19)	0.039 (2)	0.0047 (14)	−0.0057 (15)	−0.0057 (16)

### Geometric parameters (Å, °)

**Table:**

C1—F1	1.324 (5)	C7—C8	1.406 (6)
C1—F2	1.325 (5)	C8—C9	1.380 (6)
C1—F3	1.352 (5)	C8—H8	0.9500
C1—C2	1.521 (6)	C9—O4	1.284 (5)
C2—O1	1.252 (5)	C9—C10	1.499 (6)
C2—C3	1.402 (6)	C10—H10A	0.9800
C3—C4	1.388 (6)	C10—H10B	0.9800
C3—H3	0.9500	C10—H10C	0.9800
C4—O2	1.261 (5)	Co01—O3	2.032 (3)
C4—C5	1.534 (6)	Co01—O4	2.045 (3)
C5—F4	1.300 (5)	Co01—O5	2.052 (3)
C5—F6	1.321 (5)	Co01—O1	2.063 (3)
C5—F5	1.334 (6)	Co01—O2	2.064 (3)
C6—C7	1.501 (6)	Co01—O4^i^	2.122 (3)
C6—H6A	0.9800	O4—Co01^i^	2.122 (3)
C6—H6B	0.9800	O5—H5A	0.83 (4)
C6—H6C	0.9800	O5—H5B	0.84 (2)
C7—O3	1.274 (5)		
			
F1—C1—F2	107.6 (4)	O4—C9—C8	125.2 (4)
F1—C1—F3	106.5 (4)	O4—C9—C10	116.0 (4)
F2—C1—F3	106.4 (4)	C8—C9—C10	118.9 (4)
F1—C1—C2	110.1 (4)	C9—C10—H10A	109.5
F2—C1—C2	112.5 (4)	C9—C10—H10B	109.5
F3—C1—C2	113.3 (4)	H10A—C10—H10B	109.5
O1—C2—C3	128.6 (4)	C9—C10—H10C	109.5
O1—C2—C1	113.3 (4)	H10A—C10—H10C	109.5
C3—C2—C1	118.1 (4)	H10B—C10—H10C	109.5
C4—C3—C2	122.6 (4)	O3—Co01—O4	90.52 (12)
C4—C3—H3	118.7	O3—Co01—O5	99.03 (13)
C2—C3—H3	118.7	O4—Co01—O5	91.97 (12)
O2—C4—C3	128.8 (4)	O3—Co01—O1	83.91 (12)
O2—C4—C5	114.1 (4)	O4—Co01—O1	174.10 (12)
C3—C4—C5	117.1 (4)	O5—Co01—O1	90.82 (12)
F4—C5—F6	108.5 (4)	O3—Co01—O2	92.44 (12)
F4—C5—F5	106.8 (4)	O4—Co01—O2	89.58 (11)
F6—C5—F5	105.1 (4)	O5—Co01—O2	168.41 (12)
F4—C5—C4	113.9 (4)	O1—Co01—O2	88.73 (12)
F6—C5—C4	112.2 (4)	O3—Co01—O4^i^	170.59 (12)
F5—C5—C4	109.8 (4)	O4—Co01—O4^i^	80.47 (12)
C7—C6—H6A	109.5	O5—Co01—O4^i^	84.06 (12)
C7—C6—H6B	109.5	O1—Co01—O4^i^	105.00 (11)
H6A—C6—H6B	109.5	O2—Co01—O4^i^	84.87 (11)
C7—C6—H6C	109.5	C2—O1—Co01	124.6 (3)
H6A—C6—H6C	109.5	C4—O2—Co01	124.7 (3)
H6B—C6—H6C	109.5	C7—O3—Co01	125.7 (3)
O3—C7—C8	124.3 (4)	C9—O4—Co01	125.1 (3)
O3—C7—C6	116.5 (4)	C9—O4—Co01^i^	134.2 (3)
C8—C7—C6	119.2 (4)	Co01—O4—Co01^i^	99.53 (12)
C9—C8—C7	128.0 (4)	Co01—O5—H5A	117 (4)
C9—C8—H8	116.0	Co01—O5—H5B	123 (3)
C7—C8—H8	116.0	H5A—O5—H5B	118 (5)
			
F1—C1—C2—O1	−77.7 (5)	C3—C4—O2—Co01	−7.2 (7)
F2—C1—C2—O1	42.3 (6)	C5—C4—O2—Co01	170.3 (3)
F3—C1—C2—O1	163.1 (4)	O3—Co01—O2—C4	−71.2 (3)
F1—C1—C2—C3	101.1 (5)	O4—Co01—O2—C4	−161.7 (3)
F2—C1—C2—C3	−138.9 (4)	O5—Co01—O2—C4	100.5 (7)
F3—C1—C2—C3	−18.1 (6)	O1—Co01—O2—C4	12.7 (3)
O1—C2—C3—C4	0.2 (7)	O4^i^—Co01—O2—C4	117.8 (3)
C1—C2—C3—C4	−178.4 (4)	C8—C7—O3—Co01	−12.1 (6)
C2—C3—C4—O2	−2.4 (8)	C6—C7—O3—Co01	168.2 (3)
C2—C3—C4—C5	−179.9 (4)	O4—Co01—O3—C7	12.0 (3)
O2—C4—C5—F4	154.2 (4)	O5—Co01—O3—C7	104.1 (3)
C3—C4—C5—F4	−27.9 (6)	O1—Co01—O3—C7	−166.0 (3)
O2—C4—C5—F6	30.5 (6)	O2—Co01—O3—C7	−77.6 (3)
C3—C4—C5—F6	−151.7 (4)	C8—C9—O4—Co01	4.9 (6)
O2—C4—C5—F5	−86.0 (5)	C10—C9—O4—Co01	−175.4 (3)
C3—C4—C5—F5	91.8 (5)	C8—C9—O4—Co01^i^	169.5 (3)
O3—C7—C8—C9	4.8 (7)	C10—C9—O4—Co01^i^	−10.8 (6)
C6—C7—C8—C9	−175.6 (4)	O3—Co01—O4—C9	−8.5 (3)
C7—C8—C9—O4	−0.8 (7)	O5—Co01—O4—C9	−107.5 (3)
C7—C8—C9—C10	179.5 (4)	O2—Co01—O4—C9	84.0 (3)
C3—C2—O1—Co01	11.1 (6)	O4^i^—Co01—O4—C9	168.8 (4)
C1—C2—O1—Co01	−170.2 (3)	O3—Co01—O4—Co01^i^	−177.30 (13)
O3—Co01—O1—C2	78.1 (3)	O5—Co01—O4—Co01^i^	83.64 (13)
O5—Co01—O1—C2	177.1 (3)	O2—Co01—O4—Co01^i^	−84.87 (12)
O2—Co01—O1—C2	−14.5 (3)	O4^i^—Co01—O4—Co01^i^	0.0
O4^i^—Co01—O1—C2	−98.8 (3)		

Symmetry codes: (i) −*x*, −*y*, −*z*+1.

### Hydrogen-bond geometry (Å, °)

**Table:**

*D*—H···*A*	*D*—H	H···*A*	*D*···*A*	*D*—H···*A*
O5—H5A···O2^i^	0.83 (4)	2.25 (3)	2.973 (4)	147 (5)
O5—H5B···O3^ii^	0.84 (2)	1.87 (2)	2.703 (4)	169 (5)

Symmetry codes: (i) −*x*, −*y*, −*z*+1; (ii) −*x*+1, −*y*, −*z*+1.

## References

[bb1] Bruker (2008). *APEX2*, *SAINT* and *SADABS* Bruker AXS Inc., Madison, Wisconsin, USA.

[bb2] Condorelli, G. G., Motta, A., Bedoya, C., Di Mauro, G. P. & Smecca, E. (2007). *Inorg. Chim. Acta*, **360**, 170–178.

[bb3] Cotton, F. A. & Elder, R. C. (1966). *Inorg. Chem.* **5**, 423-429.

[bb4] Fahlmen, B. D. (2006). *Curr. Org. Chem.* **10**, 1021–1033.

[bb5] Hunter, G. O., Zeller, M. & Leskiw, B. D. (2009*a*). *Acta Cryst.* E**65**, m24. 10.1107/S1600536808037963 PMC2967872 21581498

[bb6] Hunter, G. O., Zeller, M. & Leskiw, B. D. (2009*b*). *Acta Cryst.* E**65**, m221–m222. 10.1107/S1600536809001846 PMC2968228 21581813

[bb7] Lerach, O. J. & Leskiw, B. D. (2008). *Rapid Commun. Mass Spectrom.* **22**, 4139–4146. 10.1002/rcm.3846 19021135

[bb8] Lerach, J. O., Zeller, M. & Leskiw, B. D. (2007). *Acta Cryst.* E**63**, m2639.

[bb9] McCann, M., Townsend, S., Devereux, M., McKee, V. & Walker, B. (2001). *Polyhedron*, **20**, 2799–2806.

[bb10] Reichert, C. & Westmore, J. B. (1969). *Inorg. Chem.* **8**, 1012–1014.

[bb11] Sheldrick, G. M. (2008). *Acta Cryst.* A**64**, 112–122. 10.1107/S0108767307043930 18156677

[bb12] Silvennoinen, R. J., Jylha, O. J. T., Lindblad, M., Sainio, J. P., Puurunen, R. L. & Krause, A. O. I. (2007). *Appl. Surf. Sci.* **253**, 4103–4111.

[bb13] Westmore, J. B. (1976). *Chem. Rev.* **6**, 695–715.

